# Association of triglyceride glucose index levels ​​with calcification patterns and vulnerability of plaques: an intravascular ultrasound study

**DOI:** 10.1007/s10554-023-02932-9

**Published:** 2023-09-29

**Authors:** Da Yin, Minxian Wang, Xuesong Liu, Weili Pan, Yongkui Ren, Jinqiu Liu

**Affiliations:** Department of Cardiology, the First Affiliated Hospital of Dalian Medical University, No. 222 Zhongshan Road, Zhongshan District, Dalian, Liaoning Province China

**Keywords:** Acute coronary syndrome, Insulin resistance, TyG index, Coronary calcification, Intravascular ultrasound, Atherosclerosis

## Abstract

Purpose: High triglyceride glucose (TyG) index level is one of the risks for cardiovascular events. The purpose of this research was to examine the correlation of the triglyceride glucose (TyG) index levels with plaque characteristics and calcification types determined by intravascular ultrasound (IVUS) in acute coronary syndrome (ACS) patients. Methods: A total of 234 acute coronary syndromes (ACS) participants who completed intravascular ultrasound (IVUS) and coronary angiography (CAG) were finally enrolled. Results: Logistic regression analysis manifested that the TyG index was independently correlated with the occurrence of coronary calcification, minimum lumen area (MLA) ≤ 4.0 mm², plaque burden (PB) > 70%, and spotty calcification. Taking the lowest group as a reference, the risk of coronary calcification (OR, 2.57; 95%CI, 1.04–6.35; *p* = 0.040), MLA ≤ 4.0 mm² (OR, 7.32; 95%CI, 2.67–20.01; *p* < 0.001), PB > 70% (OR, 2.68; 95%CI, 1.04–6.91; *p* = 0.041), and spotty calcification (OR, 1.48; 95%CI, 0.59–3.71; p = 0.407) was higher in the highest TyG index group. TyG index was converted into a dichotomous variable or a continuous variable for analysis, and we found that a similar result was observed. In addition, optimal predictive models consisting of clinical variables and the TyG index distinctly improved the ability to predict the prevalence of coronary calcification and MLA ≤ 4.0 mm² (*p* < 0.05). Conclusion: The TyG index may serve as a potential predictor for calcification patterns and plaque vulnerability.

## Introduction

Insulin resistance (IR) is a crucial intermediate process of type 2 diabetes mellitus (T2DM) and metabolic disorders, which has been verified to be closely linked to the occurrence and development of coronary atherosclerosis (AS) [1, 2]. In recent years, the TyG index as a new biomarker, calculated from ln [fasting triglycerides (mg/dL) × fasting blood glucose (mg/dL)/2], has been recognized as a practical and reliable alternative biomarker for evaluating IR due to its advantages in the simple calculation method, low cost, timing saving, and popularization [3]. Previous research has demonstrated that a high level of the TyG index is a risk for the occurrence of coronary artery calcification (CAC) and cardiovascular events [4–7]. However, the effect of the TyG index on calcification types and plaque vulnerability remains unclear. So far, no studies have been designed to investigate this question.

Intravascular ultrasound (IVUS), as an invasive imaging technique performed in the vessels, can accurately identify the shape and type of calcification in coronary artery plaques. According to several IVUS studies, spotty calcification, thin-cap fibroatheroma (TCFA), MLA ≤ 4.0 mm², and PB > 70% are strongly associated with unstable plaques [8–13]. The rupture of vulnerable plaques has been widely recognized as the primary cause of ACS and sudden cardiac death [14]. Therefore, timely identification of vulnerable plaques, search for new biomarkers, and intervention treatment is of vital significance for the prevention of ACS, which is also a research hotspot in recent years.

The present research aimed to examine the correlation of the TyG index levels with the plaque characteristics and calcification types in culprit lesions of ACS patients using IVUS.

## Methods

### Study population

**Fig. 1:**
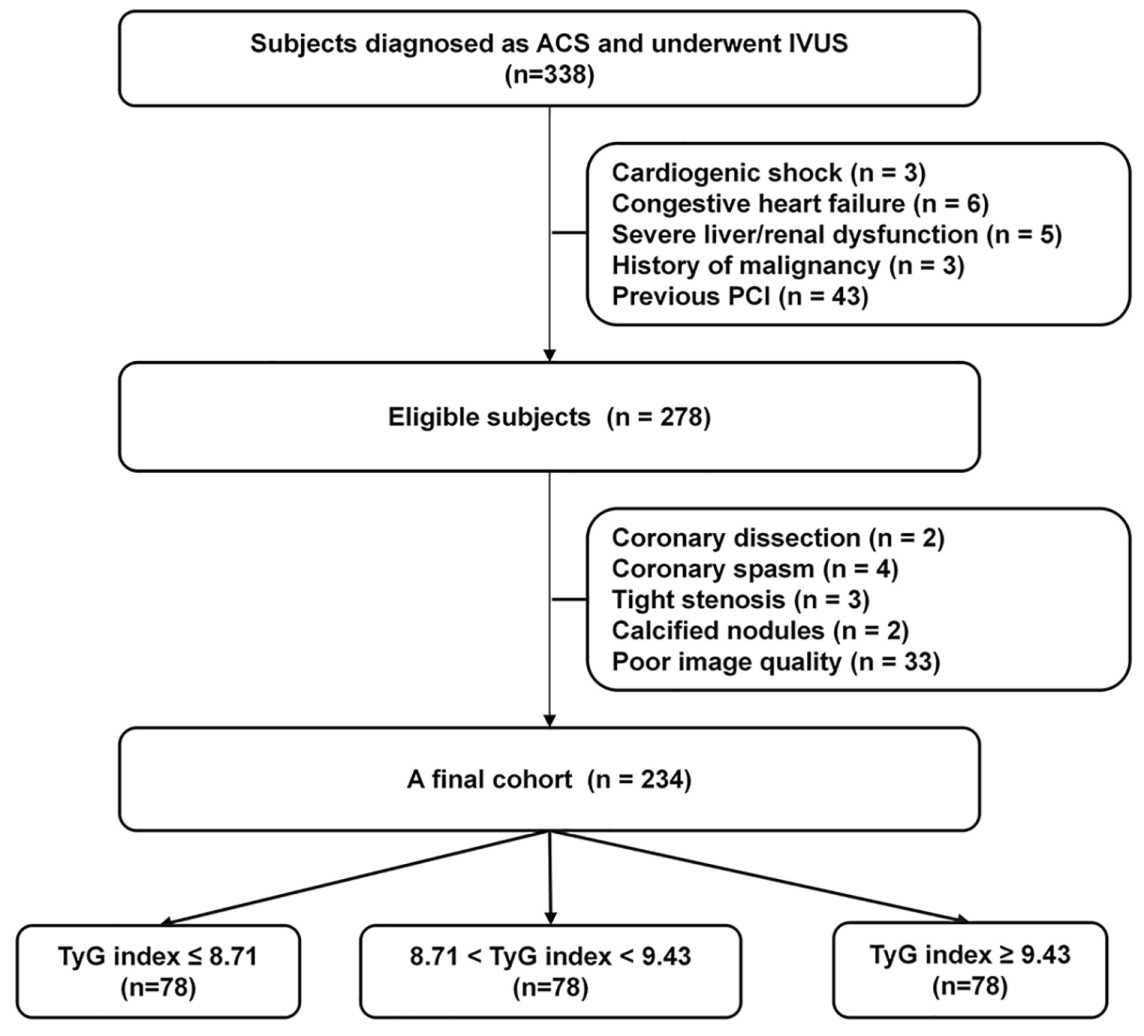
Flow chart of study. ACS, acute coronary syndrome; PCI, percutaneous intervention

This was a cross-sectional and observational study. Data from the 1st Affiliated Hospital of Dalian Medical University was recorded and statistically analyzed. 338 patients diagnosed with ACS who underwent CAG and IVUS were screened between February 2017 to July 2018. Finally, 104 subjects were excluded and 234 subjects were analyzed (a detailed flow chart was shown in Fig. 1). Furthermore, all subjects were stratified into three groups based on the TyG index levels. Due to the retrospective nature of the study, it was exempt from medical ethics based on the First Affiliated Hospital of Dalian Medical University Ethics Committee.

### Clinical and biochemical data

Demographic, medical history, and laboratory data were systematically collected. Medical history included a history of hypertension, diabetes, smoking, stroke, and old myocardial infarction (OMI). All measurements including fasting plasma glucose (FPG), lipid profile, renal function markers, and liver enzyme levels were recorded in the morning after 8 h of fasting. The blood pressure was measured in the brachial artery of the right arm using an automatic sphygmomanometer with appropriate cuff width after a 5-minute rest, and other data were reported by the patient or according to the medical records.

### Definitions

Patients were diagnosed with hypertension if they met one of the following conditions: systolic blood pressure ≥ 140mmHg or diastolic blood pressure ≥ 90mmHg at rest, previous diagnosis of hypertension, or use of antihypertensive medications. Diabetes was defined if the patient developed FPG ≥ 7.0mmoL/L within two days, history of diabetes, or used anti-diabetic treatment. Smoking was defined as currently smoking or using cigarettes regularly in the last six months. TyG index was calculated from ln [fasting triglyceride (mg/dL) × FPG (mg/dL)/2].

### Coronary angiography image acquisition

Coronary angiography was performed following the latest guidelines. Before surgery, patients were given oral aspirin (at least 100 mg/d). Heparin, which was used throughout the operation, was given to prevent thrombosis. Coronary angiography was performed by experienced operators, and coronary angiography indicators were recorded in detail. The extent of lumen stenosis and severity of coronary artery calcification was assessed on account of previous studies [15]. The segment of the culprit lesions was located by electrocardiogram and coronary angiography results.

### Intravascular ultrasound image obtainment

**Fig. 2:**
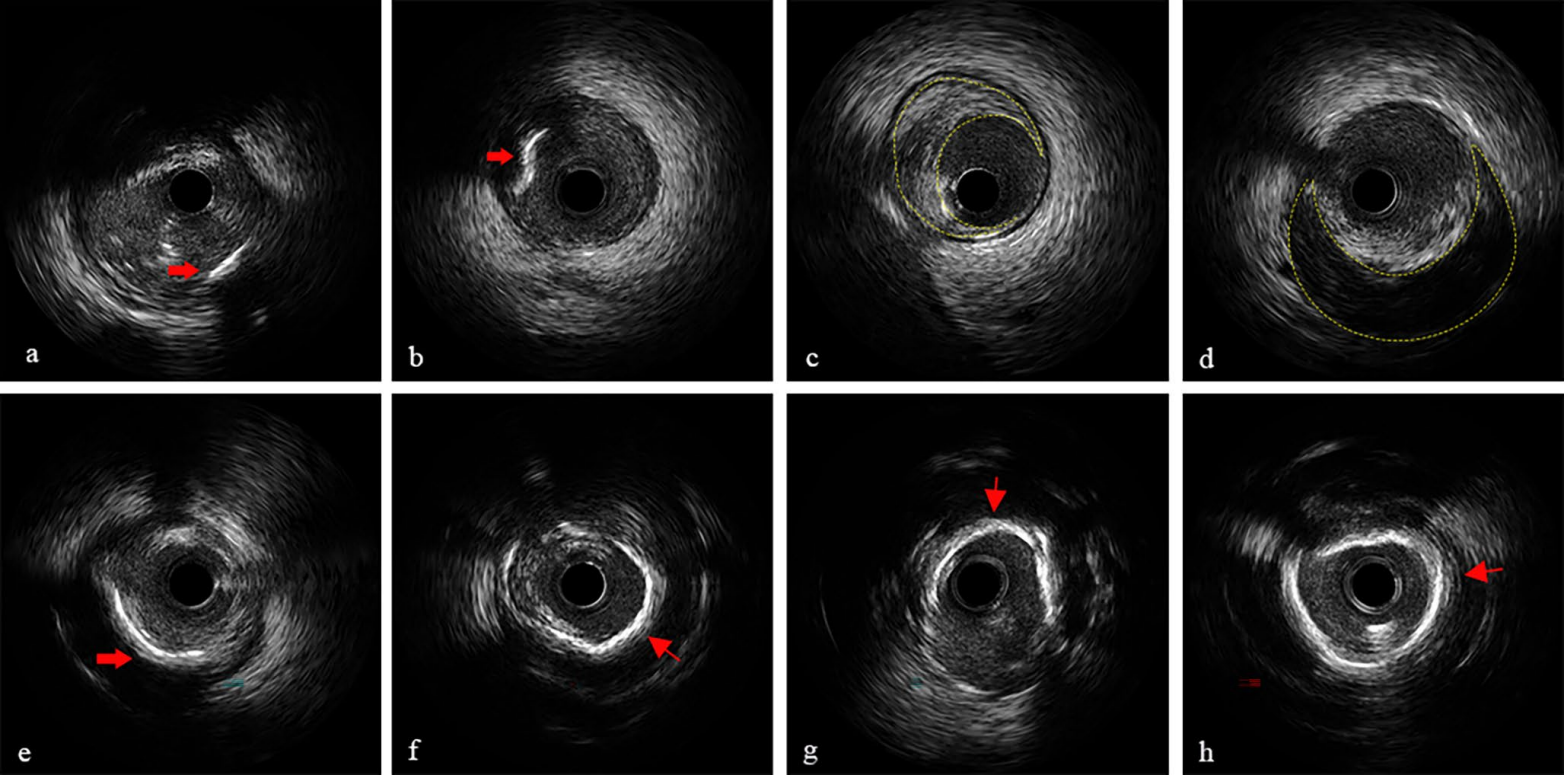
Representative IVUS images (**a**) Spotty calcification. (**b**) Calcification nodal. (**c**) PB > 70%. (**d**) Lipid-rich plaque. (**e**) Calcification with arc = 90°. (**f**) Calcification with arc = 180°. (**g**) Calcification with arc = 270°. (**h**) Annular calcification IVUS, intravascular ultrasound; PB, plaque burden

IVUS images were obtained using a commercial IVUS system (iLAB, Boston Scientific Corporation). A 40 MHz, 2.6 F imaging catheter (Atlantis SR Pro or Pro2, Boston Scientific) was advanced 15 mm distal to the culprit lesions and automatically withdrawn at 0.5 mm/s until the aortic ostium was reached. IVUS images were then archived onto CD-ROM or DVD-ROM for offline analysis. All IVUS image analyses were performed using professional offline measurement software (CASS, PIE Medical, Maastricht, the Netherlands). Qualitative and quantitative IVUS grayscale analysis per 1 mm lesion was performed according to the criteria of the American College of Cardiology Clinical Expert Consensus Document on Standards for Acquisition, Measurement and Reporting of Intravascular Ultrasound Studies [16]. IVUS images were interpreted by two experienced IVUS technicians on the CASS workstation, who were unaware of the participant’s clinical data, laboratory data, and angiographic results before processing. Another technician resolved any inconsistencies between the two technicians. The properties of the plaques are as follows: [1] lipid plaques: anechoic or hypoechoic plaques, which were darker than the external elastic membrane (external elastic membrane, EEM) as a reference.; [2] fibrous plaque: moderate echogenic plaque between anechoic lipid plaque and hyperechoic calcified plaque; [3] calcified plaque was defined as an area with a high echoic front compared to the outer membrane, visually highlighted (brighter than the outer membrane), and accompanied by acoustic shadows behind. The extent of calcification was consisting of none, spotty (arc ≤ 90°), mild (91 < arc ≤ 180°), moderate (181 < arc ≤ 270°), and severe (271 < arc ≤ 360°) calcification. Vulnerable plaques were defined as plaques with a lipid angle > 180° and a fibrous cap thickness < 65 μm. Plaque area was defined as the cross-sectional area (CSA) of EEM-lumen CSA. Plaque burden (%) was measured by reference (EEM CSA-luminal CSA) /EEM CSA×100%. MLA was defined as the luminal area at the narrowest point within the vessel where the culprit lesion is located. Calcification length was defined as the distance from the beginning of calcification to the end of calcification on the longitudinal axis of IVUS. Calcification angle was defined as the arc of calcification in degrees per 1 mm of lesion measured through the center of the lumen using an electronic protractor. Based on the conclusions of previous IVUS studies to identify plaque characteristics, the identification indicators of vulnerable plaque in this paper can rely on spotty calcification, MLA ≤ 4.0 mm², and PB > 70% [8, 12, 13]. Several common IVUS images were shown in Fig. 2.

### Statistical analysis

The data were expressed as mean ± standard deviation (SD) for normally distributed quantitative data, and the median (interquartile range) for data-skewed variables. Categorical variables were presented as numbers (percentages). To compare the differences among groups stratified by the TyG index, a one-way ANOVA or rank-sum test was used for continuous variables, and the Chi-square test or Fisher’s exact test was used for categorical variables. The correlations of TyG index levels with calcification, MLA ≤ 4.0 mm², PB > 70%, and spotty calcification were analyzed by univariate and multivariate logistic regression analysis while adjusting for potential clinical confounders. The maximum of the Yoden index ([sensitivity + specificity-1]) was used as the cut-off value to calculate the optimum cut-off for calcification, MLA ≤ 4.0 mm², PB > 70%, and spotty calcification. Moreover, the TyG index was divided into binary variables according to the cut-off value and incorporated into the model respectively. The regression results were based on the T1 group with the lowest TyG index as a reference to compare the odds ratio (OR) and 95% confidence interval (CI). In addition, to estimate the predictive ability of the TyG index for coronary calcification, MLA ≤ 4.0 mm², PB > 70%, and spotty calcification in the three different prediction models, the area under the curve (AUC) was calculated through the receiver operating characteristic (ROC) curve analysis. It should be made clear that model 1 included age, sex, history of hypertension, diabetes, current smoking, OMI, TC, and LDL-C, model 2 includes model 1 plus TyG index as a continuous variable, and model 3 includes model 1 plus TyG index as a category variable. All statistical analyses were conducted using SPSS (SPSS version 25.0; IBM, NY). A two-sided *p*-value < 0.05 was regarded as statistical significance in this study.

## Results

### Baseline characteristics

The baseline clinical characteristics of the subjects were shown in Table 1. The mean age of the 234 participants (184 men, 78.6%) was 61.2 ± 9.7 years. The incidence of hypertension, diabetes, current smoking, and OMI were 59.0%, 29.9%, 39.3%, and 18.8%, respectively. The levels of uric acid (UA), FPG, total cholesterol (TC), triglyceride (TG), LDL-C, HDL-C, and LPA as well as the prevalence of diabetes and OMI, showed significant differences among the groups. The diagnosis, medication state, and other laboratory data were not markedly different among the three groups.

**Table 1:** Clinical characteristics of subjects

		Tertiles of TyG index			
Variables	Total(n = 234)	T1(n = 78)	T2(n = 78)	T3(n = 78)	*p*
Age, years	61.2 ± 9.7	62.6 ± 9.6	59.9 ± 9.0	61.0 ± 10.3	0.212
Male, n (%)	184(78.6)	63(80.8)	64(82.1)	57(73.1)	0.335
Diagnosis, n (%)					0.265
UA	146(62.4)	54(69.2)	42(53.8)	50(64.1)	0.130
NSTEMI	56(23.9)	13(16.7)	24(30.8)	19(24.4)	0.118
STEMI	32(13.7)	11(14.1)	12(15.4)	9(11.5)	0.776
Risk factors, n (%)					
Hypertension	138(59.0)	44(56.4)	44(56.4)	50(64.1)	0.529
Diabetes mellitus	70(29.9)	15(19.2)	12(15.4)	43(55.1)	< 0.001
Current smoke	92(39.3)	30(38.5)	32(41.0)	30(38.5)	0.931
OMI	44(18.8)	23(29.5)	14(17.9)	7(9.0)	0.005
Previous stroke	8(3.4)	4(5.1)	0(0.0)	4(5.1)	0.126
Laboratory data					
Cre, umol/L	71.0(58.8–80.3)	73.0(62.8–81.0)	71.0(61.0–78.0)	66.0(56.0–88.0)	0.468
eGFR, mL/min	98.5(84.3–117.0)	94.7(82.9-111.9)	104.0(82.8-117.4)	103.8(79.6-117.8)	0.279
Uric acid, umol/L	363.6 ± 102.3	335.9 ± 94.5	375.0 ± 96.3	381.7 ± 111.4	0.011
FPG, mmol/L	5.3(4.7–6.6)	4.9(4.5–5.4)	5.1(4.6–5.9)	7.2(5.5–10.8)	< 0.001
TC, mmol/L	4.6 ± 1.1	4.3 ± 0.9	4.8 ± 1.1	4.8 ± 1.1	0.002
TG, mmol/L	1.8(1.1–2.4)	1.1(0.9–1.4)	1.9(1.5–2.3)	3.2(0.7–4.7)	< 0.001
HDL-C, mmol/L	1.1(0.9–1.3)	1.2(1.0-1.4)	1.0(0.8–1.3)	1.0(0.9–1.2)	< 0.001
LDL-C,mmol/L	2.6 ± 1.0	2.3 ± 0.7	2.8 ± 0.8	2.7 ± 0.8	< 0.001
LPa, mg/L	141.0(68.6–292.0)	164.0(98.5-343.5)	185.0(63.0-303.0)	90.0(46.4–169.0)	< 0.001
ALT, U/L	23.0(16.0–36.0)	22.0(16.0-33.8)	24.0(18.0–36.0)	22.0(16.0–41.0)	0.318
AST, U/L	22.0(17.0-34.5)	21.5(17.0–33.0)	25.0(17.0–33.0)	21.0(17.0–36.0)	0.734
Medication, n (%)					
Aspirin	232(99.1)	78(100.0)	77(98.7)	77(98.7)	> 0.99
Clopidogrel	180(76.9)	54(69.2)	65(83.3)	61(78.2)	0.107
Ticagrelor	44(18.8)	19(24.4)	8(10.3)	17(21.8)	0.056
Statins	226(96.6)	76(97.4)	74(94.9)	76(97.4)	0.736
ACEI/ARB	190(81.2)	59(75.6)	64(82.1)	67(85.9)	0.254

Note: Data are expressed as mean ± SD, median (interquartile range), or number (percentage)

Abbreviations: ALT, alanine aminotransferase; AST, aspartate aminotransferase; ACEI/ARB: angiotensin-converting enzyme inhibitor/angiotensin receptor blocker; Cre, creatinine; FPG, fasting plasma glucose; GFR, glomerular filtration rate; HDL-C, high-density lipoprotein cholesterol; LDL-C, low-density lipoprotein cholesterol; OMI, old myocardial infarction; NSTEMI, non-ST segment elevation myocardial infarction; STEMI, ST-segment elevation myocardial infarction; TC, total cholesterol; TG, triglyceride; UA, unstable angina

### Angiographic and IVUS imaging analyses

The data of angiographic and IVUS were indicated in Table 2. Significant differences in culprit arteries among the three groups were observed. (*p* = 0.005). IVUS data revealed that the occurrence of calcified angle ≤ 90°, 91–180°, 181–270°, and 271–360° was 33.3%, 17.9%, 6.0%, and 8.5%, respectively. The incidence of calcified angle ≤ 90° has marked differences among the three groups (*p* = 0.004). In addition, the incidence of coronary calcification assessed by CAG was 15.4%, but evaluated by IVUS was 65.8%. The incidence of MLA ≤ 4.0 mm² (59.0% vs.67.9% vs.83.3%, *p* = 0.004), PB > 70% (62.8% vs.79.5% vs.83.3%, *p* = 0.007), and the level of plaque area (*p* = 0.006) increased markedly with the increasing of the TyG index.

**Table 2:** Angiographic and IVUS analyses

	Total(n = 234)	T1(n = 78)	T2(n = 78)	T3(n = 78)	*p*
Culprit related artery					0.005
LM	158(67.5)	52(66.7)	63(80.8)	43(55.1)	0.003
LAD	22(9.4)	3(3.8)	6(7.7)	13(16.7)	0.019
LCX	32(13.7)	15(19.2)	4(5.1)	13(16.7)	0.024
RCA	22(9.4)	8(10.3)	5(6.4)	9(11.5)	0.521
CAG calcium	36(15.4)	16(20.5)	10(12.8)	10(12.8)	0.307
IVUS data					
calcium	154(65.8)	45(57.7)	54(69.2)	55(70.5)	0.178
calcium length, mm	4.0(0.0-10.7)	5.0(0.0–20.0)	4.0(1.0–9.0)	3.8(0.0-9.3)	0.736
Calcium angel					0.026
0°	80(34.2)	33(42.3)	24(30.8)	23(29.5)	0.178
1–90°	78(33.3)	18(23.1)	37(47.4)	23(29.5)	0.004
91–180°	42(17.9)	14(17.9)	12(15.4)	16(20.5)	0.706
181–270°	14(6.0)	4(5.1)	3(3.8)	7(9.0)	0.477
271–360°	20(8.5)	9(11.5)	2(2.6)	9(11.5)	0.069
Plaque morphology					
MLA ≤ 4.0 mm²	164(70.1)	46(59.0)	53(67.9)	65(83.3)	0.004
PB > 70%	176(75.2)	49(62.8)	62(79.5)	65(83.3)	0.007
plaque area, mm²	11.6(8.5–14.7)	11.2(7.8–13.6)	11.3(8.5–14.0)	12.9(10.7–16.2)	0.006

Note: Data are expressed as median (interquartile range), or number (percentage)

Abbreviations: CAG, coronary angiography; IVUS, Intravascular ultrasound; LM, left main; LAD, left ascending artery; LCX, left circumflex artery; MLA, minimal lumen area; PB, plaque burden; RCA, right coronary artery

### Association of the TyG index levels with calcification patterns and plaque characteristics

As shown in Table 3, the results from the binary logistic regressions manifested that the TyG index was independently correlated with the occurrence of coronary artery calcification, MLA ≤ 4.0 mm², PB > 70%, and spotty calcification. After adjustments for clinical confounders including age, sex, history of hypertension, diabetes, smoking, OMI, TC levels, and LDL-C levels, these relationships remained significant (*p* < 0.05) (Model 2). But for spotty calcification, only the results in the second group were statistically significant. Taking the group with the lowest TyG index as a reference, the risk of coronary calcification (OR, 2.57; 95%CI, 1.04–6.35; *p* = 0.040), MLA ≤ 4.0 mm² (OR, 7.32; 95%CI, 2.67–20.01; *p* < 0.001), and the PB > 70% (OR, 2.68; 95%CI, 1.04–6.91; *p* = 0.041) was higher in the group with the highest TyG index. The optimal cut-off values were determined by the receiver operating characteristic (ROC) curve, the TyG index was converted into a dichotomous variable or a continuous variable for analysis, and similar results were obtained.

**Table 3:** Association of TyG index for calcification types and plaque characteristics

	TyG index	Model I OR (95%CI)	*p*	Model II OR (95%CI)	*p*
Calcification	T1	1		1	
	T2	1.65(0.86–3.19)	0.136	2.47(1.12–5.37)	0.026
	T3	1.75(0.90–3.40)	0.096	2.57(1.04–6.35)	0.040
	aTyG index	1.58(1.06–2.35)	0.025	1.88(1.15–3.08)	0.013
	bTyG index	3.42(1.81–6.44)	< 0.001	4.24(1.98–9.07)	< 0.001
Spotty calcification	T1	1		1	
	T2	3.01(1.51–5.99)	0.002	2.50(1.16–5.39)	0.019
	T3	1.39(0.68–2.86)	0.364	1.48(0.59–3.71)	0.407
	aTyG index	1.28(0.86–1.89)	0.220	1.49(0.90–2.46)	0.125
	bTyG index	3.50(1.67–7.35)	0.001	3.39(1.53–7.51)	0.003
MLA ≤ 4.0 mm²	T1	1		1	
	T2	1.48(0.77–2.84)	0.245	1.99(0.91–4.35)	0.085
	T3	3.48(1.65–7.34)	0.001	7.32(2.67–20.01)	< 0.001
	aTyG index	2.85(1.79–4.54)	< 0.001	4.84(2.63–8.94)	< 0.001
	bTyG index	3.35(1.87–6.01)	< 0.001	5.46(2.59–11.49)	< 0.001
PB > 70%	T1	1		1	
	T2	2.29(1.12–4.69)	0.023	2.10(0.95–4.67)	0.067
	T3	2.96(1.40–6.28)	0.005	2.68(1.04–6.91)	0.041
	aTyG index	2.38(1.48–3.81)	< 0.001	2.48(1.44–4.25)	0.001
	bTyG index	3.20(1.73–5.91)	< 0.001	3.30(1.61–6.75)	0.001

Note: The cut-off values of TyG index for calcification, spotty calcification, MLA ≤ 4.0 mm², and PB > 70% were 8.465 mg/dL, 8.565 mg/dL, 8.885 mg/dL, and 8.825 mg/dL, respectively

Model I: unadjusted for none

Model II: adjusted for age, sex, hypertension, diabetes, current smoking, OMI, TC, and LDL-C.

aTyG index was analyzed as a continuous variable

bTyG index was analyzed as a category variable according to the cut-off value determined by ROC.

Abbreviations: CI, confidence interval; MLA, minimal lumen area; OR, odds ratio; PB, plaque burden; ROC, receiver operating characteristic curve

### An optimal predictive model based on the TyG index

**Fig. 3:**
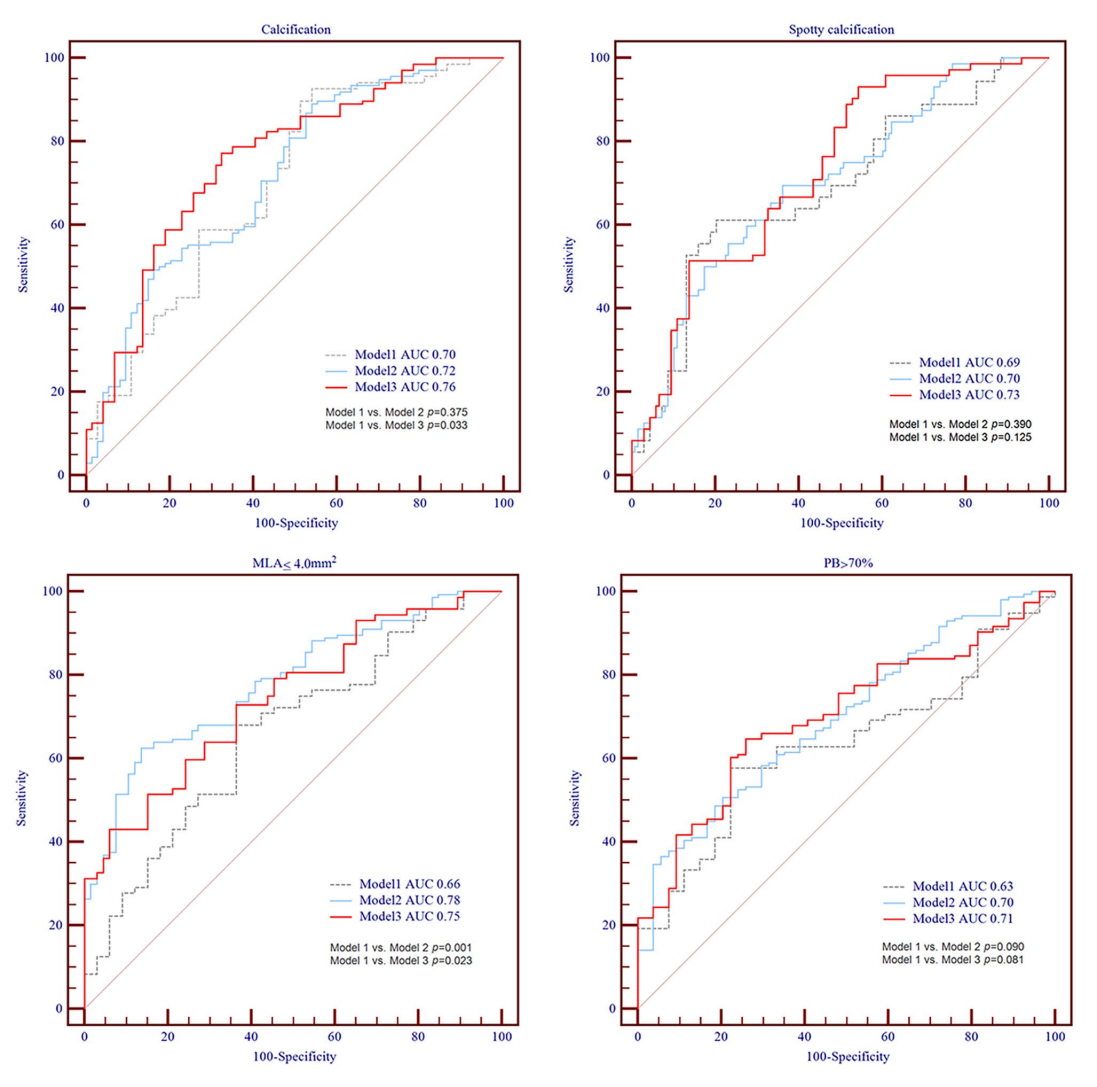
AUCs for the presence of calcification, spotty calcification, MLA ≤ 4.0 mm², and PB > 70%. Model 1: adjusted for age, sex, hypertension, diabetes, current smoking, OMI, TC, and LDL-C. Model 2: model 1 plus TyG index as a continuous variable. Model 3: model 1 plus TyG index as a category variable according to cut-off values evaluated by ROC. The cut-off values of TyG index for calcification, spotty calcification, MLA ≤ 4.0 mm², and PB > 70% were 8.465 mg/dL, 8.565 mg/dL, 8.885 mg/dL, and 8.825 mg/dL, respectively. MLA, minimal lumen area; PB, plaque burden

ROC results were revealed in Fig. 3. Model 1 included age, sex, history of hypertension, diabetes, current smoking, OMI, TC, and LDL-C. The AUCs of model 1 for calcification was 0.70, spotty calcification was 0.69, MLA ≤ 4.0 mm² was 0.66, and PB > 70% was 0.63. Model 2 (model 1 plus TyG index as a continuous variable) strongly enhanced the predictive ability for the presence of MLA ≤ 4.0 mm², the AUC increased from 0.66 to 0.78 (*p* = 0.001). Moreover, the AUCs of Model 3 (model 1 plus TyG index as a category variable) had been further increased than Model 1 (calcification: 0.70 to 0.76, *p* = 0.033; MLA ≤ 4.0 mm²: 0.66 to 0.75, *p* = 0.023). Nonetheless, these models were not statistically different in predicting the occurrence of spotty calcifications and PB > 70%.

## Discussion

And for all we know, this is the research to examine the correlation of the TyG index levels with plaque characteristics and calcification patterns in ACS patients using IVUS. Our main finding was an obvious correlation of the TyG index with the emergence of coronary calcification, MLA ≤ 4.0 mm², PB > 70%, and spotty calcification, even after adjusting for clinical confounders. Additionally, the risk of coronary calcification, MLA ≤ 4.0 mm², PB > 70% crept up with the TyG index. Another essential result was that the predictive model (TyG index plus clinical risk factors) was superior to other models in predicting coronary artery calcification and plaque instability.

### TyG index and calcification patterns

TyG index, identified as an alternative biomarker for insulin resistance (IR), has been reported to be independently related to CAC in healthy adults [7, 17, 18]. Whereas, the correlations of the TyG index with diverse calcification patterns remain uncertain. In this study, we not only verified the correlation of the TyG index with the occurrence of CAC in ACS patients by IVUS but also further found that the TyG index was related to spotty calcification. Despite the underlying mechanism of the correlation between the TyG index and coronary calcification not being elucidated, it is reasonable to speculate that insulin resistance may play a pivotal role, which is consistent with previous reports.

IR was reported to be independently correlated with CAC [19, 20]. The mechanism is that IR leads to vascular damage in vivo by causing vascular REDOX dysfunction, thereby boosting the risk of vascular disease. Vascular damage caused by insulin resistance can be separated into two categories: functional and structural, involving impaired vasodilation mediated by chemical mediators, decreased compliance of the vascular wall (arterial stiffness), the enhanced thickness of the arterial wall, and vascular calcification [21]. Furthermore, IR can promote the formation and progression of atherosclerosis by down-regulating insulin receptor-mediated signaling pathways in vascular intima cells [22, 23].

Previous research has recorded that the TyG index was closely correlated with cardiovascular events or poor prognosis [4, 24]. Nevertheless, the exact mechanism remains uncertain. Fortunately, our research can partially explain this phenomenon. We have verified that the TyG index was independently correlated with spotty calcification in the current study, implying that it has some value in predicting unstable lesions. At the same time, inspired by a pathology-based study, the stability of the lesion depended on the degree and type of calcification. Among them, lesions that appeared densely calcification on the image may be stable plaques (fibrocalcific plaque), while those related to early or unstable plaques were micro, spotty, and sheet-like calcifications [8, 25, 26]. Most importantly, spotty calcifications were more common in ACS than in stable angina [8]. In a word, high levels of the TyG index represent the vulnerable plaques and high-risk individuals to some extent. Unfortunately, in the predictive models incorporating clinical factors and the TyG index, there was no obvious prediction power for the occurrence of spotty calcification. It may be due to the limited sample size of this research, so further research was needed.

### TyG index and plaque characteristics

As the main parameters of IVUS, MLA ≤ 4.0 mm², PB > 70%, and TCFAs were used to assess the plaque characteristics, plaque burden, and plaque vulnerability [27]. In the current research, we found that the TyG index was markedly correlated with the occurrence of MLA ≤ 4.0 mm² and PB > 70%. Several related researchers support our results to some extent.

As shown in the formula based on the TyG index, we can infer that the TyG index level is positively correlated with both plasma glucose levels and serum triglyceride (TG) levels. As far as we know, diabetic patients have a higher prevalence of plaque rupture [5], higher lipid burden, larger maximal lipid arc, and a higher incidence of thin-cap fibroatheroma (TCFA). An OCT-based study confirmed an increased incidence of TCFAs and lipid-rich plaques in patients with elevated serum triglyceride levels [28]. Furthermore, an IVUS study demonstrated that coronary plaque volume changes increased with the increase of serum TG levels [29]. In addition, a previous study has revealed that the amount of coronary calcification was positively associated with plaque burden [30]. Meanwhile, the independent correlation between TyG index levels and coronary calcification was obvious in our research. Therefore, the positive relationships between the TyG index and plaque burden were well-founded. Unfortunately, there was not sufficient evidence in this study to discuss the correlation between the TyG index level and TCFA. This may be partly due to the influence of IVUS pixel gray value on resolution, which ultimately leads to the wrong identification of TCFAs.

### Limitation

First of all, our study only involved participants from a single center. Given that triglyceride levels varied somewhat by race. Therefore, it was necessary to test whether the TyG index is an appropriate biomarker of CAC and plaque vulnerability in other populations. Second, the subject’s nutritional data was not recorded; therefore, we cannot rule out the potential influence of nutritional habits on blood triglycerides. Third, the TyG index was assessed only once after admission. There were limited data on the dynamics of TyG index levels in the subsequent course of ACS. Fourth, the reason for the high proportion of left main disease was that left main lesions were more suitable for IVUS to guide PCI. Finally, since this is a cross-sectional survey, we were unable to establish a causal relationship between the TyG index and vulnerable plaque. The significance of the TyG index in predicting adverse cardiovascular outcomes also needs to be explored, so further prospective studies are warranted.

## Conclusion

A higher TyG index level was markedly correlated with the incidence of spotty calcification, MLA ≤ 4.0 mm², and PB > 70%. According to the results of this study, the TyG index may serve as a non-invasive potential biomarker for the clinical prediction of calcification patterns and plaque features that may represent unstable plaques. This is effective for preventing secondary acute cardiovascular events caused by vulnerable plaques in advance.

## Abbreviations

TyG: Triglyceride glucose
IR: Insulin resistance
ACS: Acute coronary syndrome
CAC: Coronary artery calcification
IVUS: Intravascular ultrasound
MLA: Minimum lumen area
PB: Plaque burden
TG: Triglyceride
LDL-C: Low-density lipoprotein cholesterol
